# Multifunctional Tannic Acid and Polyamino Acid Layer-by-Layer Coatings for Tailored Implant Surfaces

**DOI:** 10.1021/acsbiomaterials.5c00832

**Published:** 2025-08-19

**Authors:** Enrique Oreja, Daria Zaytseva-Zotova, Agnes Rogala, Alejandro Barrantes, Phillip B. Messersmith, Hanna Tiainen

**Affiliations:** † Department of Biomaterials, 6305 University of Oslo, PO Box 1109, Blindern, 0317 Oslo, Norway; ‡ Oral Research Laboratory, University of Oslo, PO Box 1109, Blindern, 0317 Oslo, Norway; § Departments of Bioengineering and Materials Science and Engineering, 1438 University of California, Berkeley, 210 Hearst Mining Building, Berkeley, California 94720, United States; ∥ Materials Sciences Division, Lawrence Berkeley National Laboratory, 1 Cyclotron Road, Berkeley, California 94720, United States

**Keywords:** QCM-D, layer-by-layer, tannic acid, poly-l-lysine, poly-l-arginine

## Abstract

Polyphenols are attractive candidates for biological surface modifications due to their adhesive, antioxidant, anti-inflammatory, and antimicrobial properties. However, the uncontrolled release of polyphenols, such as tannic acid (TA), from the surface may lead to adverse biological responses. Polyamino acids (PAAs), such as poly-l-lysine (PLL) and poly-l-arginine (PLR), improve wound healing and act as antimicrobial agents, but their high positive charge can result in cytotoxicity. In this study, TA and PAAs were combined in layer-by-layer (LbL) coatings to take advantage of the beneficial biological properties of these molecules while limiting their release and the potential damage they can cause to the surrounding tissues. Coating formation was monitored using a quartz crystal microbalance with dissipation. Linear growth of the TA thickness versus time was observed for most of the studied conditions. TA/PLR coatings resulted in higher dissipation shifts compared with TA/PLL, indicating that PLR results in more viscoelastic coatings. Dissipation progressively increased with the number of deposited bilayers, the increase being more noticeable for TA/PLR. Modeled thickness of the coatings was greater for TA/PLR than for TA/PLL coatings. The interaction between TA and PAAs in the LbL coatings was found to be noncovalent, as determined by Fourier transform infrared (FTIR) and Ultraviolet–visible (UV–vis) spectroscopy. The addition of PAAs to the coatings prevented the release of TA but reduced their antioxidant capacity. Human gingival fibroblasts (hGFs) showed a higher viability on TA/PLL than on TA/PLR coatings. Cells adhered to both multilayer types and formed multiple focal adhesions (FA), which are essential for proper cell function.

## Graphical abstract

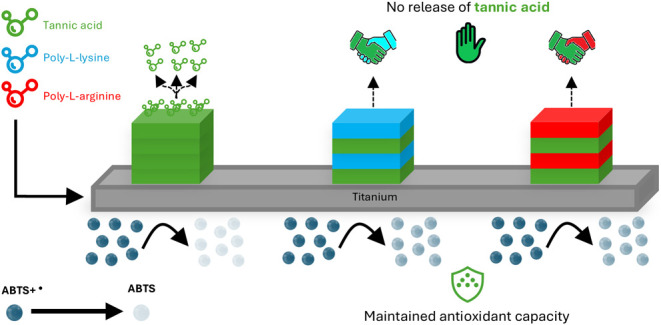

## 1 Introduction

Surface modifications of implants are essential for improving their integration within biological tissues, enhancing their biocompatibility, mitigating infections and promoting tissue regeneration.^1,2^ Surface modifications can be categorized as physical, chemical or biological.^3^ Physical modifications produce changes in the surface topography and roughness by applying treatments such as laser, plasma spraying, or sandblasting, while chemical modifications change the chemical properties of the surface, such as sol–gel or anodic oxidation processes. Lastly, biological/biochemical surface modifications include bioactive molecules such as proteins or peptides with the aim of enhancing the interaction with the biological environment.^4,5^

Polyphenols have emerged as potential candidates for biological/biochemical surface modifications due to their antioxidant, anti-inflammatory, and antimicrobial properties.^6,7^ Among polyphenols, tannic acid (TA) stands out as a versatile molecule that can adhere to different substrates and form coatings. TA is a plant-derived polyphenol composed of a central unit of glucose and an average of ten gallic acid units attached to it.^8^ The presence of hydroxyl groups and aromatic rings makes TA an excellent candidate for noncovalent interactions with different molecules such as polysaccharides, proteins, or peptides.^9–11^ TA is highly soluble in water with a phenolic p*K*_a_ close to 8.5.^12^ In acidic conditions, the phenolic groups of TA are protonated and act as a hydrogen donor, while when the pH conditions are basic, the hydroxyl groups are deprotonated and act as hydrogen acceptors, allowing TA to interact with other molecules via hydrogen bonding.^13^ Furthermore, oxidation processes at basic pH, result in the formation of semiquinones and quinones, which can react with nucleophiles, such as thiols or amines, via Schiff base formation or Michael addition.^14^ Due to the amphoteric properties of TA, the working pH will strongly affect the interaction with other molecules. Additionally, the phenolic rings allow TA to interact via π-π stacking and metal complexation to form metal-phenolic networks (MPNs).^12^

TA-MPN networks had been thoroughly studied previously.^15–17^ The design of TA-MPNs coatings is carried out by making use of metallic ions from transition metals, such as Fe^3+^, Cu^2+^ or Mn^2+^.^18^ Nevertheless, the coating formation is limited to a monolayer due to the fast reaction between TA and the transition metals, instantly precipitating and inhibiting the continuous deposition.^19,20^ Weber et al. suggested an alternative to TA-MNP networks, investigating the role of orthosilicic acid (Si_aq_) in coating formation. They went beyond the monolayer limitation, achieving continuous deposition and precise control over the layer formation.^21,22^ Despite the additional control afforded by this approach, a high concentration of TA and a noncontrolled release from the surface can result in adverse biological conditions.^23,24^ Therefore, there is a need to improve the performance of TA coatings by achieving a controlled release and an increased cell response.

Layer-by-layer (LbL) technique is commonly used in controlled drug delivery systems or coatings with antimicrobial properties.^25–28^ LbL assemblies are based on the electrostatic interactions between oppositely charged molecules, offering high control and simplicity of the system.^29^ TA has been previously used in LbL build-ups, acting as the negatively charged part of the system.^30,31^ We hypothesize that the addition of a positively charged polyamino acid (PAA) to the coating will stabilize the deposited layer and prevent uncontrolled release of TA. Both poly-l-lysine (PLL) and poly-l-arginine (PLR) are positively charged PAAs that have been used in LbL coatings.^32–36^ It has been shown that the presence of PLL or PLR alone or in LbL coatings can improve cell viability of implant surfaces by interacting with the negatively charged cell membrane components^37^ and can promote cell adhesion to the biomaterial.^38–40^

Understanding the interactions between TA-PLL and TA-PLR and how their individual properties are affected when combined in LbL coatings is key to optimizing TA-based coatings. In this present study, we aim to achieve precise control over the coating formation on Titanium substrates by means of Quartz crystal microbalance with dissipation (QCM-D). Additionally, we support our results by studying the interaction between molecules using spectroscopy and evaluating the stability and antioxidant properties of the coatings. Finally, we studied cell responses to the coatings by confocal microscopy and other cell studies.

## 2 Materials and Methods

### 2.1 Materials

Tannic acid (ACS grade, MW = 1701.2 g/mol; LOT#MKBN9606 V), poly-l-arginine (PLR; MW = 15–70 kDa), poly-l-lysine (PLL; MW = 15–30 kDa), sodium metasilicate pentahydrate (95%) and HEPES (BioPerformance, ≥99.5%), bovine serum albumin (BSA; cold ethanol fraction, pH 5.2 ≥ 96.0%), Dulbecco’s modified Eagle’s medium (DMEM), fetal bovine serum (FBS), phosphate-buffered saline (PBS), Tween 20, Triton X-100, 0.02% trypsin-EDTA solution, and mouse monoclonal antibody to vinculin were obtained from Sigma-Aldrich. Secondary antibodies Alexa Fluor 488 goat antimouse IgG, Alexa Fluor 568 Phalloidin, and alamarBlue Cell Viability Reagent were supplied by Invitrogen. DAPI was purchased from Thermo Fisher Scientific. Penicillin–streptomycin and GlutaMAX supplements were obtained from Gibco. Cytotoxicity Detection Kit (LDH) was supplied by Roche.

### 2.2 Coating Formation

#### 2.2.1 Quartz Crystal Microbalance with Dissipation Monitoring (QCM-D)

A quartz crystal microbalance with dissipation monitoring **(**QSense E4, Biolin Scientific) was used to monitor real-time adsorption and the stability of the layer-by-layer coating (LbL). Titanium (Ti) sensors (QSX 310, Biolin Scientific) were used to monitor changes in frequency (Δ*F*) and dissipation (Δ*D*) for the fundamental frequency (∼5 MHz) and the third, fifth, seventh, ninth, and 11th harmonics under different experimental conditions. For the sake of clarity, only the first three overtones are plotted. Sensors were cleaned according to the manufacturer’s protocol before and after each experiment. The mentioned protocol includes 15 min ultraviolet/ozone (UV/O_3_) treatment (Novascan PSD-UV4), two subsequent sonication steps in 2% sodium dodecyl sulfate (SDS) and in Milli-Q H_2_O, and a final step consisting of 15 min treatment in UV/O_3_. Prior to the measurements, the sensors were equilibrated with coating buffer containing 0.1 M HEPES and 0.6 M NaCl adjusted to pH 6.8 at 21 °C and 0.1 mL/min until a baseline was reached. Tannic acid (TA), poly-l-lysine (PLL), and poly-l-arginine (PLR) were dissolved in coating buffer. TA concentration was 1 mg/mL, while PLL and PLR were prepared at a concentration of 10 mg/mL in Milli-Q water and then diluted to 0.05 mg/mL in coating buffer. 80 μM sodium silicate was added to all TA solutions immediately before each TA layer deposition.^22^ A washing step with coating buffer was included after every deposited layer to remove loosely bound molecules from the surface. TA coating time varied between 10, 30, and 60 min, while PLL/PLR (0.05 mg/mL) coating times were kept constant at 5 min. Each bilayer consisted of the alternate deposition between TA and PLL or PLR, and the number of bilayers varied between 5, 3, and 2 for TA coating times of 10, 30, and 60 min, respectively. All of the experiments were performed in triplicate (*n* = 3).

The thickness of the deposited LbL coatings was calculated with QTools Software (Biolin Scientific, Version 3.1.33). As each deposited bilayer showed Δ*D* > 1 × 10^–6^, the obtained data from QCM-D was analyzed according to Voigt–Kelvin model using water density and viscosity values.^41^ Modeling was conducted by using the first three overtones. Furthermore, the physical thickness of the LbL coatings deposited on silicon wafers was measured by using atomic force microscopy (AFM; MFP 3D, Asylum Research). For this purpose, half of the silicon wafer was coated following the protocol described for titanium coins (coated surface), while the other half of the wafer was masked during the coating deposition using polyvinylsiloxane, which was removed once the coating deposition was complete (noncoated surface). Following removal of the silicone mask, the samples were scanned in 3 nonoverlapping spots using an AC240TS cantilever in contact mode at a scan angle of 90°, a scan rate of 1 Hz, a set point of 0.5 mV, and a scan-area of 90 × 90 μm^2^. Coating thicknesses were determined by averaging the height difference between the coated and noncoated surfaces of three extracted cross-section profiles with a width of 10 pixels and are given as mean ± standard deviation.

#### 2.2.2 Titanium Coins

LbL multilayers were also deposited on Ti coins (Ø = 6 mm) by alternately dipping them in solutions containing 1 mg/mL TA, 0.05 mg/mL PLL, and 0.05 mg/mL PLR with a washing step in coating buffer in between. All of the solutions were prepared in coating buffer as previously described for QCM-D analysis. Prior to the coating, Ti coins were first cleaned by dipping them subsequently in 50% nitric acid (HNO_3_), 40% sodium hydroxide (NaOH), 2% SDS, and Milli-Q water. A final step of 15 min in a UV/O_3_ chamber was included. Ti coins were immersed in the coating solution in 6-well plates and placed on a rocking platform at constant movement with a speed of 30 rpm. 1 mL/coin was used for the layer-by-layer deposition. The LbL coating consisted of 5 bilayers. Coating time for TA was 30 min, while PLL/PLR deposition time was 5 min. The TA monolayer was included as a reference. For the TA monolayer, 2.5 mL/coin of 1 mg/mL TA solution was used, and the coating time was adjusted to be equal to the LbL deposition (∼4 h). Ti coins were dipped in Milli-Q H_2_O after the last deposited layer and dried using a filter paper. The coins were stored at room temperature and used for ABTS, Prussian blue, and cell assays.

### 2.3 Spectroscopy

#### 2.3.1 Fourier Transform Infrared (FTIR) Spectroscopy

To identify the main functional groups in the deposited coatings, FTIR spectra were recorded with a PerkinElmer Spectrum 400 using a universal attenuated total reflection (ATR) sampler with a resolution of 2 cm^–1^ and 32 scans. The interaction between TA-PLL and TA-PLR was elucidated by preparing dry precipitates as follows: TA 5 mg/mL, PLL 0.25 mg/mL, and PLR 0.25 mg/mL were dissolved in coating buffer, and reaction mixtures composed of TA and PLL and TA and PLR solutions were incubated overnight at room temperature. Subsequently, the solutions were centrifuged for 5 min at 14,800 rpm to sediment the formed TA-PAA precipitates. Once the supernatant had been removed, the TA-PAA precipitates were rinsed three times in 1 mL Milli-Q water to rinse off the remaining salts. Lastly, the samples were centrifuged under vacuum conditions (Savant SpeedVac DNA110, Thermo Scientific) for 2 h to dehydrate the samples before FTIR measurements. TA, PLL, and PLR powders were used as reference spectra, directly obtained from the commercial reagents. The obtained data were processed in Origin 2022 (OriginLab). Spectra were normalized prior to identifying the main functional groups and changes in their wavenumber.

#### 2.3.2 Ultraviolet–Visible (UV–Vis) Spectroscopy

UV–vis spectra of the TA monolayer and the TA-PLL and TA-PLR multilayers coated on quartz slides were obtained using a Lambda 25 UV–vis spectrophotometer (PerkinElmer). The spectra were recorded between 450 and 200 nm, with a resolution of 1 nm. Quartz slides were coated with TA, TA-PLL, and TA-PLR following the protocol previously described for titanium coins.

### 2.4 Surface Characterization

#### 2.4.1 Surface Zeta Potential

The surface zeta potential (ζ) of the TA monolayer and TA-PLL and TA-PLR multilayers coated on Si wafers was determined in saline buffer containing 2 mM sodium phosphate and 10 mM NaCl. Measurements were conducted at a conductivity of 0.5 mS/cm using a Nano ZS Zetasizer (Malvern Panalytical) and negatively charged COOH-terminated polystyrene tracer particles (Figure S1; 100 nm, 250 μg/mL; micromod Partikeltechnologie GmbH). The applied voltage was set to 10 V. The Si wafers were coated according to the protocol previously described for titanium coins.

#### 2.4.2 Antioxidant Capacity

To assess the antioxidant capacity of the coated surfaces, an ABTS assay was performed.^42^ A reaction mixture was prepared by combining 7 mM ABTS with 2.45 mM K_2_(SO_2_)_2_ in a 1:1 ratio. This mixture was then incubated overnight in the dark to create the ABTS^+●^ radicals. Prior to the assay, the reaction mixture was diluted in Milli-Q water to adjust the absorption at 690 nm to 1.0 for 200 μL in a 96-well plate. Six coated Ti coins per well were placed in a 6-well plate and incubated in the ABTS solution at 1 mL/coin at room temperature on a rocking platform at a speed of 30 rpm and protected from light. 200 μL aliquots were taken at different time points from each well, and their absorbance at 690 nm was measured immediately using a microplate reader (BioTek ELx800). After each measurement, the aliquots were returned to the well. The measurements were performed in triplicate, ABTS solution in the absence of Ti coins was used as a control, and bare Ti coins without TA were included as a reference for no radical scavenging.

#### 2.4.3 Release of Polyphenols

To measure the amount of TA released from the coatings, a Prussian blue assay was conducted.^43^ Milli-Q water was filtered three times through activated coal to remove iron contaminants. Six coated Ti coins per well were placed in a six-well plate and incubated in 1 mL/coin of Milli-Q water at room temperature on a rocking platform at a speed of 30 rpm and protected from light. 150 μL aliquots were extracted from each well at different time points and transferred into a 96-well plate. To each well of the 96-well plate, 25 μL of 20 mM FeCl_3_ solution dissolved in 0.1 M HCl was added, followed by 25 μL of 16 mM K_4_Fe­(CN)_6_. Following 5 min of incubation at room temperature, absorbance at 690 nm was measured using a microplate reader (BioTek ELx800). TA concentration was calculated from a standard curve obtained by measuring the absorbance for freshly prepared serial dilutions of TA solution. The measurements were performed in triplicate, and uncoated Ti coins were used as a control for no TA release.

### 2.5 Cell Response

#### 2.5.1 General Culture Conditions

Primary human gingival fibroblasts (hGFs), purchased from the American Type Culture Collection (ATCC), were routinely cultured in Dulbecco’s Modified Eagle’s Medium (DMEM), containing 1 g/L glucose, 10% fetal bovine serum (FBS), 100 U/mL of penicillin, 100 μg/mL streptomycin, and 2 mM GlutaMAX. Cells were maintained at a constant temperature of 37 °C within a humidified atmosphere containing 5% CO_2_ and subcultured prior to reaching confluence by using trypsin-EDTA solution.

#### 2.5.2 Cell Growth on Ti Coins

Polished titanium (Ti) coins (Ø = 6 mm) were cleaned and aseptically coated with TA and PLL/PLR as described previously. hGFs were seeded onto the Ti coins at a density of 7000 cells/well (100 μL/well, serum-free DMEM) in a 96-well plate and allowed to attach for 2 h. Subsequently, 100 μL of DMEM supplemented with 20% FBS was added to each well, and cells were incubated at 37 °C in a CO_2_ incubator for 22 h before cell assays.

#### 2.5.3 Acute Cytotoxicity

To evaluate the cytotoxicity of the modified Ti surfaces on cells, a lactate dehydrogenase (LDH) assay was employed. After 24 h of cell incubation, 150 μL of cell culture medium was collected and centrifuged at 270*g* for 2 min. Then, 100 μL of the supernatant was mixed 1:1 with the reaction mixture and incubated for 30 min in the dark according to the manufacturer’s guidelines. Following the addition of stop solution to each well, the absorbance was measured at 490 nm (BioTek ELx800).

#### 2.5.4 Cell Viability

To quantitatively assess the viability of cells cultivated on the modified Ti disks, an alamarBlue assay was used following the manufacturer’s instructions. Briefly, 24 h after cell seeding, the culture medium was replaced with fresh DMEM enriched with 10% FBS and containing a 10% alamarBlue solution. Subsequently, the samples were incubated at 37 °C in a CO_2_ incubator for an additional 5 h. Finally, 150 μL of the cell culture medium was collected, and the fluorescence intensity of the alamarBlue reagent was measured at 590 nm (BioTek Synergy HT).

#### 2.5.5 Confocal Microscopy

To examine cell morphology on modified Ti surfaces, the cells were rinsed with PBS, fixed with 4% paraformaldehyde for 20 min, permeabilized with 0.1% Triton X-100 for 10 min, and subsequently blocked for 60 min in 2% BSA/PBS. The samples were then incubated with antivinculin (1:100) for 1 h. Subsequently, DNA staining was performed using DAPI (0.1 μg/mL) for 20 min in the dark. Finally, the spatial distribution of vinculin and cytoskeleton organization was visualized through a 30-min staining in the dark with secondary antibody goat antimouse Alexa Fluor 488 (1:400) and Alexa Fluor 568 Phalloidin (1:4000), respectively. Images were acquired using an upright confocal laser scanning microscope equipped with 20×/0.50 HCX APO L U–V–I and 63×/0.90 HC APO UVIS CS2 objectives.

### 2.6 Data Analysis

The software Origin version 2022 (OriginLab Corporation, Northampton, MA) was used to study the statistical differences between groups. One-way ANOVA with post hoc Tukey test was used. Data are presented as mean ± standard deviation.

## 3 Results and Discussion

### 3.1 Coating Formation

Layer-by-layer (LbL) deposition of alternating TA and PLL/PLR was studied under different experimental conditions: TA coating time 10 min with 5 bilayers (Figure 1A–D), 30 min coating with 3 bilayers (Figure 1E–H), and 60 min coating with 2 bilayers (Figures S2–S4). The TA coating time was varied to determine the effect of TA layer thickness on the LbL formation process.

**Figure 1:**
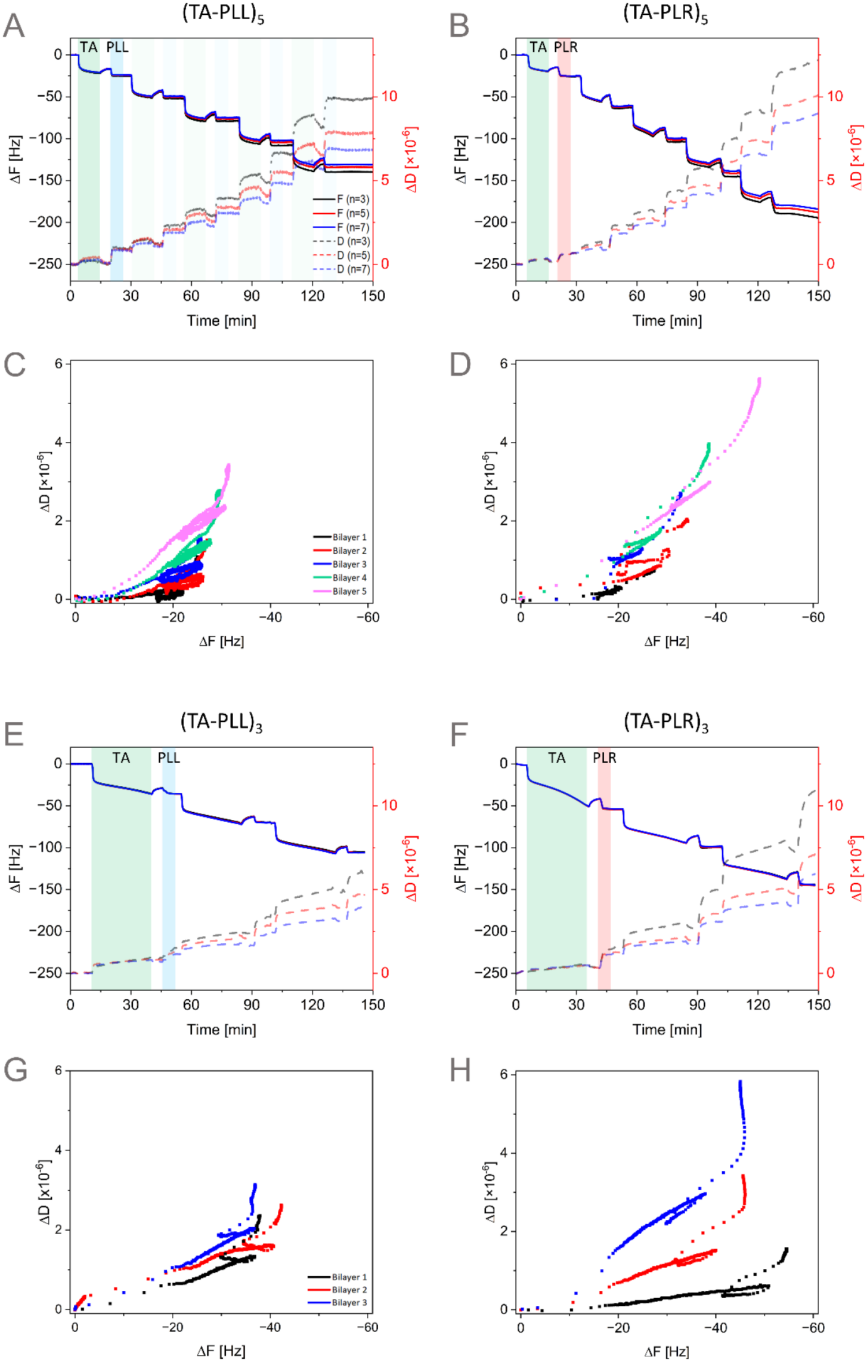
Deposition over time of TA-PLL (A, C, E, G) and TA-PLR (B, D, F, H) coatings monitored with QCM-D. Representative plots of frequency and dissipation changes at harmonics (*n*) 3, 5, and 7 over time for 5 bilayers and TA deposition time 10 min (A, B). Data were replotted as Δ*D* vs Δ*F* to obtain qualitative information about the viscoelastic properties of the coating (C, D). The same procedure was followed for TA deposition time of 30 min and 3 bilayers (E–H).

Time-dependent changes in Δ*F* and Δ*D* (Figure 1A,B,E,F) provide real-time information on the adsorption processes. Generally, a decrease in Δ*F* indicates an increase in the mass deposited on the sensor, while an increase in Δ*D* reflects a decrease in the layer rigidity.^44^ On the other hand, Δ*D*/Δ*F* plots (Figure 1C,D,G,H) are used to obtain qualitative information about the viscoelastic properties of the deposited layer, relating the changes in dissipation caused by the adsorbed mass. The slope in Δ*D* vs Δ*F* plots indicates how rigidity changes throughout the process. The steeper the slope, the more viscoelastic the layer becomes.^45^ Additionally, the density of the data points is related to the adsorption kinetics. The distance between data points on Δ*D* vs Δ*F* plots reflects how fast changes in dissipation are produced per unit of mass adsorbed (frequency changes), more distant points indicate faster kinetics.^46^

The formation of the first bilayer in the LbL deposition process begins with the adsorption of TA onto the Ti surface (Figure 1A,B, green highlights). This process is characterized by a rapid decrease in frequency, reaching ∼20 Hz within the first few minutes of deposition, as previously reported in the literature.^22,47^ This phenomenon can be observed in the Δ*D* vs Δ*F* plots (Figure 1C,D), where the spacing between data points indicates fast kinetics of the interaction, which is assumed to occur via coordination of TA with TiO­(OH) complexes.^48^ Additionally, the Δ*D* remains almost constant despite the sudden drop in Δ*F*, suggesting the formation of a very rigid layer. Thereafter, the deposition of TA on the surface continues at a slower rate, via silicate-TA cross-linking.^22^ This phenomenon is reflected in the Δ*D* vs Δ*F* plots (Figure 1C,D) by a higher density of data points, indicating slower deposition kinetics.

After 10 min deposition of the first TA layer (Figure 1A–D), buffer was introduced into the QCM-D chamber, interrupting the cross-linking process. This caused an increase in frequency due to the removal of loosely bound TA particles. This step had little impact on the viscoelastic properties of the film as the dissipation remained constant despite mass being removed from the surface, resulting in an increase in frequency.

After 5 min of rinsing with buffer, the deposition of PLL begins (Figure 1A, blue highlight). There is an immediate interaction between TA and PLL, quickly saturating the surface. This phenomenon is observed in the Δ*D* versus Δ*F* plot as scattered data points (Figure 1C). Hydrogen bonds and electrostatic forces between these molecules are likely the main driving forces for their rapid interaction.^49^ PLL was deposited for 5 min, allowing enough time to saturate the surface, reaching a plateau in Δ*F*(*t*) (Figure 1A). To remove unbound PLL, buffer was introduced into the system, concluding the formation of the first bilayer. During this step, no changes in Δ*F* are observed, indicating that the formed TA-PLL bilayer is stable in an aqueous environment. However, a vertical increase was registered in the Δ*D* vs Δ*F* plot (Figure 1C). These changes might be attributed to slow changes in the conformation of PLL.^50^

The deposition profile of the first bilayer for TA coating time 10 min was similar for both PAAs, with changes in frequency of ∼20 Hz. Both PLL (Figure 1A) and PLR (Figure 1B) saturated the surface of the sensor within 5 min of deposition and showed a similar slope in Δ*D*/Δ*F* plots (Figure 1C,D). Additionally, changes in dissipation for the first deposited bilayer were similar, indicating similarities in the rigidity of the film. Subsequent bilayers showed minimal differences from the first, although TA was deposited on PLL or PLR instead of Ti. Despite this, the deposition profiles observed in Δ*D*(*t*) and Δ*F*(*t*) were comparable to each other. Although the binding kinetics were similar throughout the multilayer buildup, as the number of bilayers increased, the changes in Δ*F* became more significant, especially for TA-PLR. Another difference observed was the change in the viscoelastic properties of the film. There was a clear increase in the slope of Δ*D* vs Δ*F*, indicating that as the number of deposited layers increased, the deposited bilayer became more dissipative than the previous one. Again, this difference was more evident for TA-PLR than for the TA-PLL multilayers.

To test the influence of TA thickness in the LbL deposition, coating time was increased from 10 to 30 min. The number of bilayers was reduced from 5 to 3 to keep the total deposition time similar. The (TA-PAA)_3_ coating (Figure 1E–H) deposition profile was comparable to that of (TA-PAA)_5_, except for the TA layer thickness. Despite the thicker layer of TA, PAAs still deposited onto the TA layer (Figure 1E,F), indicating that the thickness of the TA layer did not affect the LbL process. As observed in (TA-PAA)_5_, each subsequent bilayer became more viscoelastic. This difference was more evident again for TA-PLR (Figure 1H) than for TA-PLL (Figure 1G). This behavior highlights the different properties of the TA-PLL and TA-PLR systems depending on the number of bilayers and deposition time.

As observed in Figure 2A,D, LbL deposition occurs linearly as the number of bilayers increases, except for (TA-PLR)_5_. Instead, a nonlinear growth was observed for each deposited bilayer, which was in agreement with previous reports.^51,52^ This nonlinear deposition of the (TA-PLR)_5_ multilayers was further confirmed when Δ*F* for each individual bilayer was determined (Figure 2B). The slope of cumulative Δ*F* curves was steeper for TA-PLR than for TA-PLL, independently of the number of deposited bilayers (Figure 2A,D). Additionally, the slope for 3 deposited bilayers was also steeper compared to 5 deposited bilayers within the same PAA. This difference is attributed to the greater thickness of the TA layer. Figure 2B,E shows the thickness of each deposited bilayer. For TA-PLL this value remains constant throughout the number of deposited bilayers, confirming the linearity observed in Figure 2A,D. On the other hand, the slope increased for (TA-PLR)_5_ and appears to be constant for (TA-PLR)_3_.

**Figure 2:**
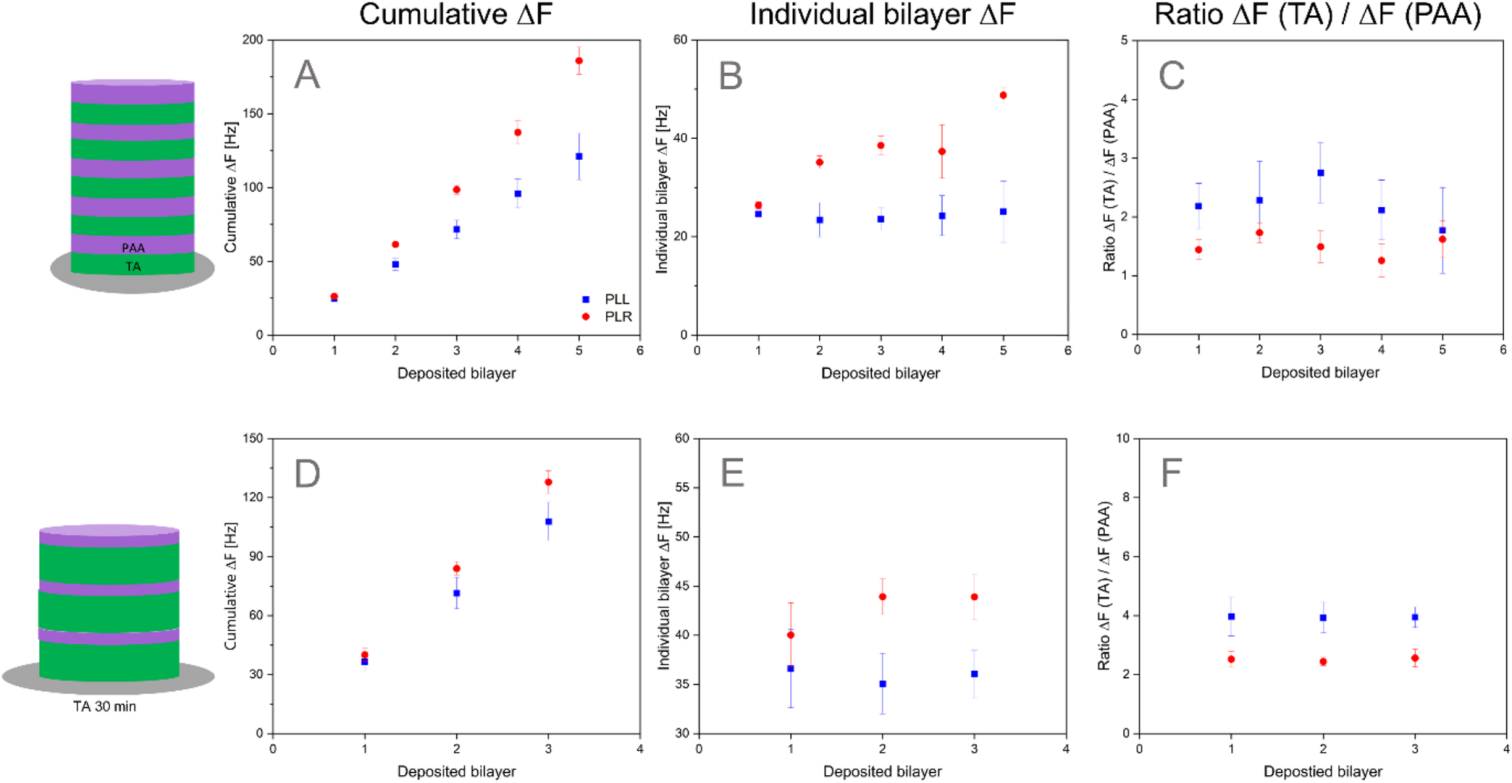
Effect of PLL and PLR on the frequency shifts during bilayer formation. Comparison of cumulative (A, D), individual (B, E), and TA/PAA ratio frequency shifts for the deposition of TA-PLL (blue) and TA-PLR (red). Panels (A–C) show data for 5 deposited bilayers, while (D–F) for 3 deposited bilayers. Results are presented as mean ± SD (*n* = 3).

The modeled thickness (Table 1) of LbL was used to support the information obtained from the plots. According to the data presented in Table 1, (TA-PLR) was thicker compared to (TA-PLL) in all tested conditions, as observed in Figure 2A,D. (TA-PLL)_3_ vs (TA-PLR)_3_ thickness was not significantly different, while (TA-PLL)_5_ vs (TA-PLR)_5_ showed statistical differences (*p* < 0.05). Additionally, the total thickness of (TA-PLL)_5_ and (TA-PLL)_3_ was found to be significantly different (*p* < 0.05), while no significant differences were found between (TA-PLR)_5_ and (TA-PLR)_3_. The thickness of each individual layer was found to be statistically similar for all (TA-PLL) conditions and for (TA-PLR)_3_, as observed in Figure 2B,D. The nonlinear behavior observed for (TA-PLR)_5_ (Figure 2A, red), was reflected during the modeling by finding statistical differences between the thickness of the first bilayer and the last two bilayers (*p* < 0.05).

**Table 1:** Modeled Thickness of TA-PAA Coatings during LbL Deposition^a^

	(TA-PLL)_5_	(TA-PLR)_5_		(TA-PLL)_3_	(TA-PLR)_3_
bilayer 1	4.4 ± 0.1 nm	4.6 ± 0.1 nm	bilayer 1	7.2 ± 0.9 nm	9.9 ± 2.3 nm
bilayer 2	3.9 ± 0.2 nm	6.7 ± 0.7 nm	bilayer 2	8.4 ± 1.1 nm	8.1 ± 0.4 nm
bilayer 3	5.7 ± 3.1 nm	7.1 ± 1.3 nm	bilayer 3	6.9 ± 1.5 nm	8.6 ± 0.3 nm
bilayer 4	3.9 ± 0.1 nm	8.7 ± 2.0 nm			
bilayer 5	7.6 ± 0.2 nm	9.3 ± 1.3 nm			
entire coating	19.7 ± 0.8 nm	37.8 ± 9.0 nm	entire coating	22.5 ± 0.5 nm	24.1 ± 1.7 nm

a Results are presented as mean ± SD (*n* = 3). The thickness is reported for each individual bilayer, as well as for the entire coating. A comparison is made between PLL and PLR, considering two different TA coating times (10 and 30 min) and two different numbers of bilayers (5 and 3).

Physical thickness of the deposited layers was measured from the AFM profiles, as presented in Figure S5. The obtained thickness was correlated with the modeled thickness obtained by QCM-D for (TA-PLL)_5_ and (TA-PLR)_5_. That same trend was not observed when the thickness was measured for (TA-PAA)_3_. The measured physical thickness was higher than the modeled thickness based on the QCM-D data. During the AFM measurements, a repulsive interaction between the cantilever and the surface was observed, with this phenomenon being more noticeable for coatings containing PLL. This effect, in addition to the uneven topography observed for the multilayers (Figure S6), may contribute to the discrepancy between the modeled thickness and the measured thickness. Furthermore, the thickness modeled based on QCM-D data considers the entire deposited layer on the surface of the QCM-D sensors, whereas the AFM measurements are determined based on three nonoverlapping areas of 90 × 90 μm^2^ at the interface of the coated and noncoated silicon wafer. Thus, the AFM thickness values are more likely to be affected by local variations in surface topography than the modeled thickness values obtained by QCM-D. Nonetheless, the same trend was observed as in QCM-D, obtaining thicker values for coatings containing PLR.

Despite the larger changes registered in the Δ*F* for PLR, the ratio between TA/PLR is smaller than that between TA/PLL (Figure 2C,F). Differences were also observed between those of (TA-PAA)_5_ and (TA-PAA)_3_. While for (TA-PAA)_5_, the ratio varies depending on the deposited bilayer, the ratio remained constant during the deposition of (TA-PAA)_3_. These observed differences are likely explained by diffusion processes, as it has been proposed that PAA chains can diffuse in and out of the multilayer.^52,53^ When a new layer of PAA is deposited onto the film, some PAA chains diffuse into the interior, acting as “free PAA chains”, interacting weakly with the previously deposited TA layers. During the buffer rinse, some of these free chains diffuse to the exterior and are removed, but due to the positive charge on the surface, a portion of them remains trapped within the film. When the next layer of TA is deposited, the TA molecules first interact with the PAAs on the surface, but the PAA chains that had previously diffused can now diffuse back out and interact with the TA.^54^ This diffusion effect is more noticeable in (TA-PAA)_5_ compared to (TA-PAA)_3_, most likely because the thinner TA layer in (TA-PAA)_5_ allows more movement between the layers, explaining why the ratio TA/PAA varies within the deposited bilayer.

It was observed in the experimental data of (TA-PLR)_5_ that Δ*F* increased for both TA and PLR every subsequent bilayer, while for (TA-PLL)_5_ Δ*F* increased only for TA, and instead decreased for PLL with every deposited layer. The difference in the adsorption behavior can be explained by the chemical and structural differences between PLL and PLR. Both PLL and PLR are assumed to bind in a flat conformation. The p*K*_a_ of PLL is around 10.6 given by its amino groups.^55^ The p*K*_a_ of PLR was assumed to be near 12.5 ^56^ due to guanidine groups,^57^ although recent studies suggest even higher values of p*K*_a_.^58^ The higher p*K*_a_ value of PLR than PLL, together with the structural differences, presence of guanidine groups in PLR versus amino groups in PLL, may lead to stronger interactions between TA and PLR. Additionally, the larger size of PLR compared to PLL results in more noticeable changes in Δ*F*.

### 3.2 Molecular Interactions between TA and PAA

#### 3.2.1 FTIR Spectroscopy

Due to the different behavior observed during QCM-D analysis for TA-PLL and TA-PLR, and to further investigate the interactions between PAA and TA, FTIR spectroscopy was performed, obtaining information on the main functional groups and peak shifts. At the working pH (6.8), the deprotonated hydroxyl groups of TA are expected to interact with amine groups from PAA; therefore, those regions in the spectra will be of interest.

The broad band observed in the TA spectrum (Figure 3, black) centered at around 3320 cm^–1^ corresponds to the O–H stretching. In that region, PLL (Figure 3A, blue) has several bands between 3300 and 3000 cm^–1^ associated with N–H stretching from the amide group^59^ and PLR (Figure 3B, blue) has two bands centered at 3274 and 3154 cm^–1^, attributed to C–H and N–H stretching.^60^ In the spectrum of the TA-PLL precipitate (Figure 3A, red), the center of the band in that region shifted from 3320 cm^–1^ in TA spectra to 3202 cm^–1^, and a shoulder emerged around 2968 cm^–1^ as a result of the presence of PLL in the precipitate. Similar changes were observed for TA-PLR (Figure 3B, red), where the band did not exhibit the characteristic shape of OH stretching, but two shoulders appeared at 3344 and 3214 cm^–1^ corresponding to PLR C–H and N–H stretching. As for the second region of interest between 1750 and 1500 cm^–1^, TA spectra (Figure 3, black) exhibited two bands centered at 1698 and 1608 cm^–1^ that correspond to the symmetrical carbonyl (C=O) stretching and the symmetrical C–O stretch, respectively.^61,62^ In the presence of PAA, the C=O stretching band did not shift. While the symmetrical C–O band shifted 4–1604 cm^–1^ for both PAA. PLL (Figure 3A, blue) and PLR (Figure 3B, blue) main bands appeared at 1644 and 1518 cm^–1^ and 1644 and 1542 cm^–1^, respectively. Those bands belong to amide I carbonyl stretching and amide II N–H bending.^63–65^ A band centered at 1504 cm^–1^ was observed in TA-PLL (Figure 3A, red), corresponding to PLL amide I carbonyl stretching, but no clear presence of the amide II N–H bending was observed in the spectrum, probably due to the overlapping of both signals. For TA-PLR (Figure 3B, red), the two characteristic bands were found in the spectra, shifting from 1644 to 1660 cm^–1^ and from 1542 to 1504 cm^–1^. Lastly, the bands centered at 1532, 1444, and 1176 cm^–1^ in TA spectra (Figure 3, black) are attributed to the aromatic C=C stretching.^61,62^ In the presence of PAA, the band at 1176 cm^–1^ shifted to 1170 cm^–1^ while the rest of the bands remained unchanged.

**Figure 3:**
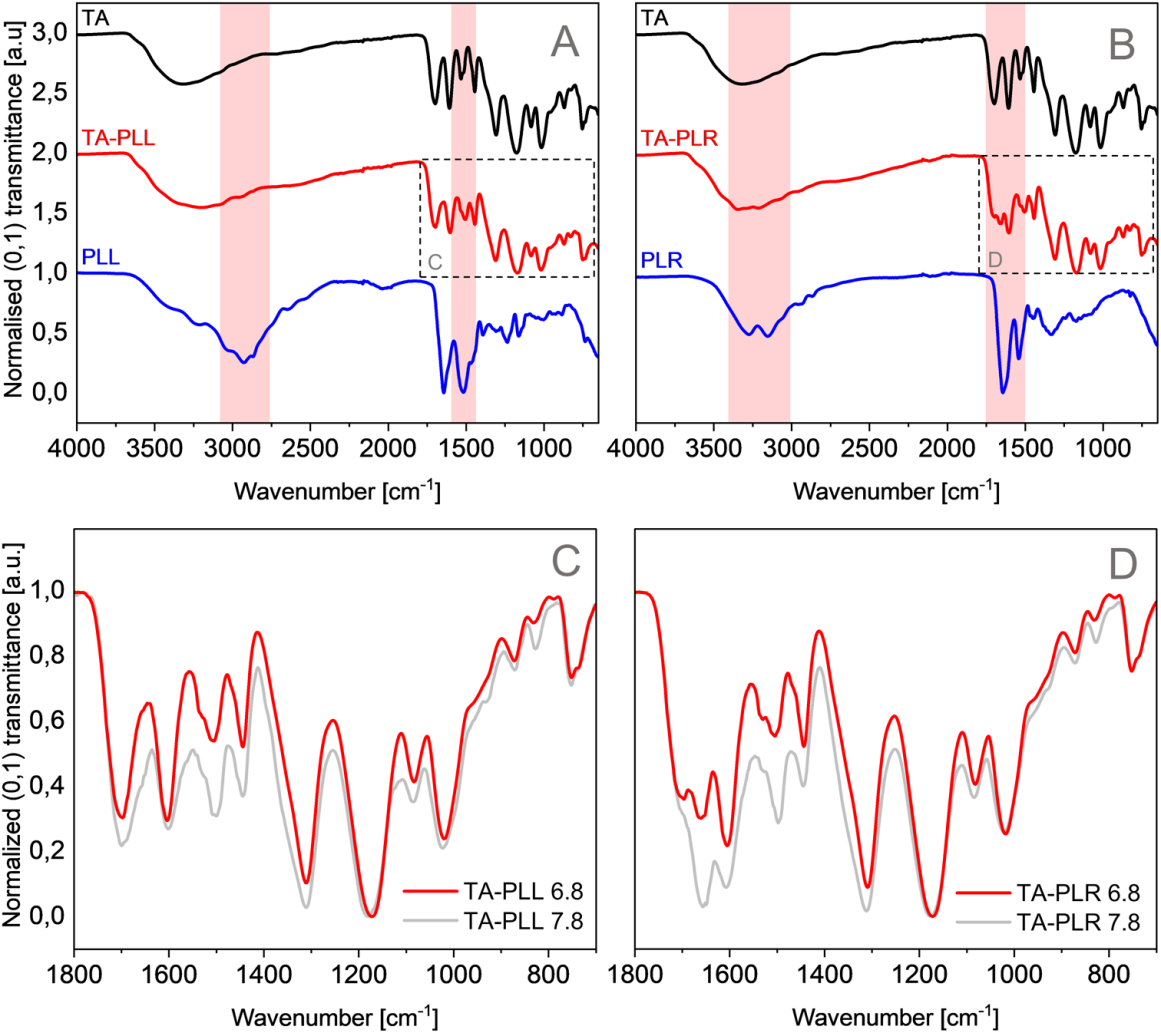
FTIR spectra of TA-PAA precipitates and commercial powders of TA and PAA. (A, B) Comparison between TA, TA-PLL, and PLL (A); and TA, TA-PLR, and PLR (B). The FTIR spectra of TA (black), PAA (blue), and TA-PAA precipitates prepared at pH 6.8 (red). The red-shaded regions highlight areas of interest where the presence of both compounds, TA and PAA, can be observed (red line). (C, D) Enlarged views of the region of interest for TA-PLL (C) and TA-PLR (D) precipitated at pH 6.8 (red) and 7.8 (gray). Spectra were additionally background corrected.

As can be seen from Figure 3C,D, the increase in pH from 6.8 to 7.8 led to an increase in peak intensities and two noticeable peak shifts. A new peak has been noticed at 1120 cm^–1^, which was more pronounced for TA-PLL (Figure 3C). These changes in spectra can be primarily attributed to the oxidation of TA at pH 7.8, leading to oxidative cross-linking reactions.^47^ Similar changes in FTIR spectra were found by Kang et al., who examined complexes formed between TA and ε-PLL at pH 5, 7, and 9.^66^ Based on an increase at both C–N (1150–1050 cm^–1^) and C=N (1700–1630 cm^–1^) bands in FTIR spectra at higher pH and the following TGA analysis, the authors suggested Schiff base formation as the primary oxidative coupling reaction at increased pH.

#### 3.2.2 UV–Vis Spectroscopy

UV–vis spectroscopy was used to further investigate the nature of the interaction between TA and PAA. Two different pH conditions were used. At pH 6.8 (Figure 4A), only the hydroxyl groups are deprotonated, while at pH 7.8 (Figure 4B), the TA molecule oxidizes into its quinone form. This difference in pH can alter the mechanism of interaction between TA and PAAs, and, therefore, it is important to investigate these interactions in both oxidative and nonoxidative conditions (Figure 4). The first band observed in the TA spectrum appears around 215 nm, corresponding to the n → π* electronic transition.^67^ The next band is a combination of two distinct peaks, one around 280 nm (λ_1_) and the other near 315 nm (λ_2_), both belonging to the π → π* electronic transitions.^67^ In its neutral form, TA has a band centered at around 283 nm. However, in solution, the TA molecule begins to deprotonate into its enolate form, shifting the band toward higher energy electronic transitions.^68^ It has been previously shown in the literature^67^ that the TA molecule is strongly affected by pH. This is the reason why two peaks were observed in the π → π* electronic transition band. The peak around 334 nm becomes visible as the deprotonation of the hydroxyl groups in the TA molecule increases with pH. This peak is not present at acidic pH but appears in more basic solutions, where TA starts oxidizing.^12^ A deconvolution was performed on the π → π* transition band to separate the two peaks (λ_1_ and λ_2_) and to determine their peak position area (A_1_ and A_2_). As shown in Table 2, the wavelength of peaks 1 and 2 for TA at both pH remained unchanged, while the ratio of the peak areas (A_2_/A_1_) for TA at pH 6.8 is 4.9, and at pH 7.8 it increases to 6.7, due to the oxidation of the TA molecule.

**Figure 4:**
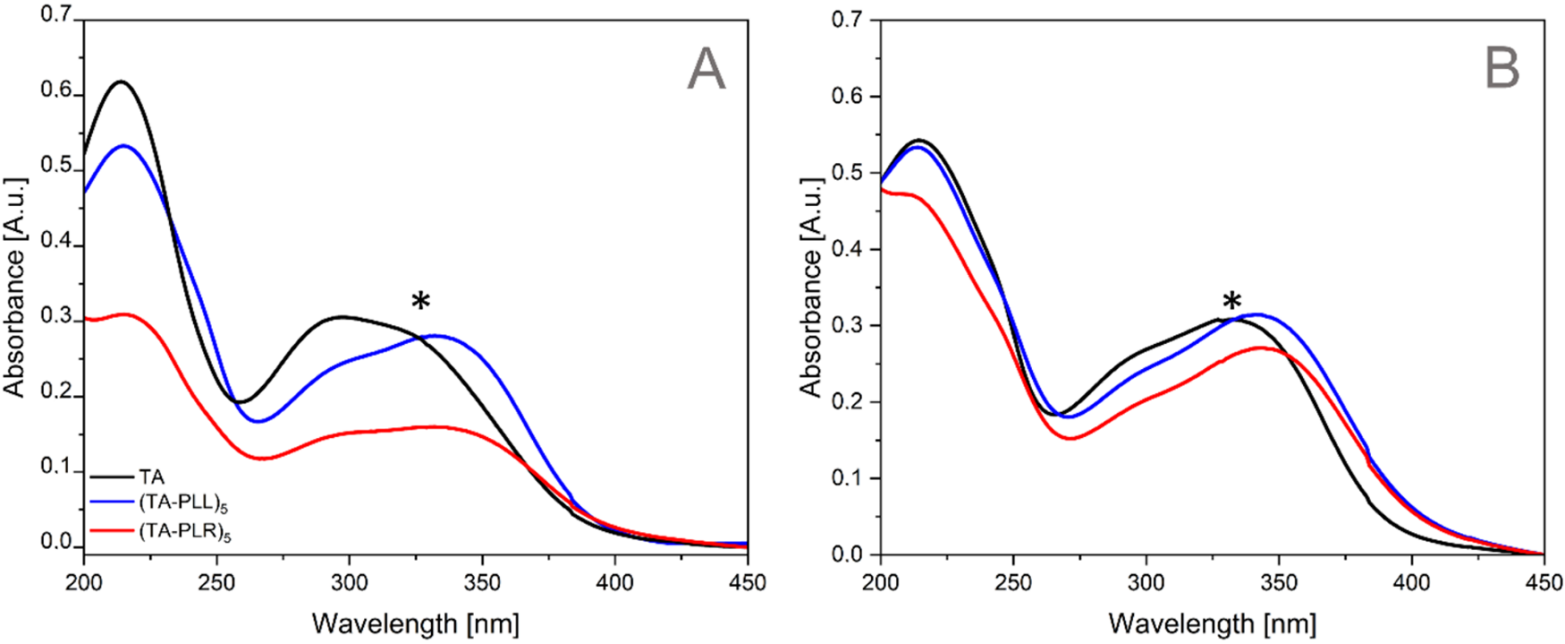
UV–vis spectra of TA (black), (TA-PLL)_5_ (blue), and (TA-PLR)_5_ (red) at pH 6.8 (A) and 7.8 (B). Two main bands are observed at 215 and 290 nm. The asterisk (*) on the graph marks the region of interest where changes can be detected due to the presence of PAA, indicating potential interactions between the compounds.

**Table 2:** Deconvolution of the Two Peaks of the π → π* Band^a^

	pH 6.8			pH 7.8		
	TA	(TA-PLL)_5_	(TA-PLR)_5_	TA	(TA-PLL)_5_	(TA-PLR)_5_
λ_1_ [nm]	277 ± 5	281 ± 10	282 ± 9	280 ± 8	286 ± 9	288 ± 9
λ_2_ [nm]	310 ± 14	321 ± 17	323 ± 18	318 ± 20	329 ± 16	331 ± 16
A_2_/A_1_	4.9 ± 1.2	5.5 ± 2.7	4.5 ± 1.8	6.7 ± 2.9	7.6 ± 3	7.7 ± 1.9

a The results are presented as the mean ± SD (*n* = 4). The table shows a comparative study of the effect of pH on TA and the influence of PAA on the UV-Vis spectra. The comparison was made focusing on the peak wavelengths (λ) and the ratio of the areas between peaks 1 and 2 (A_2_/A_1_). The study was made at two different pH (6.8 and 7.8).

The spectra of TA-PLL and TA-PLR did not show the formation of any new bands, reinforcing the possibility of an interaction of a physical nature. As reported in the literature, the amino groups present in both PAAs do not absorb in the visible region of the spectrum and only appear in the far-UV region due to carbonyl group electronic transitions.^69^ As a result, the presence of PAA in the measured samples was not evident except for PLR, for which a new peak began to appear around 200 nm (Figure 4). However, the presence of both PAAs in the coatings did alter the recorded spectra. As shown in Table 2, both deconvoluted peaks (λ_1_ and λ_2_) shifted to higher wavelengths when compared to the plain TA coatings deposited at both nonoxidative (pH = 6.8) and oxidative (pH = 7.8) conditions. This shift was more pronounced for λ_2_. Regarding the A_2_/A_1_ ratio, the presence of PAA does not alter this value compared to that of TA. Additionally, the presence of PAA does not affect the n → π* electronic transition band, as the peak position of the band remains the same as registered for TA.

The p*K*_a_ of TA is around 8.5, as previously reported in the literature.^12,70^ Near and above this pH, TA is capable of reacting with other compounds through chemical reactions such as Michael addition or Schiff base reactions.^13^ These reactions occur when amine groups are in neutral form. Considering that the working pH was several pH units below the p*K*_a_ of PAAs, the majority of PLL and PLR was protonated, suggesting that chemical cross-linking was not the driving force of the interaction. Based on the QCM-D, FTIR, and UV–vis analyses and considering the pH of the experimental conditions, the proposed interaction mechanism between TA and PAA is likely through hydrogen bonding or electrostatic forces.

### 3.3 TA Release and Antioxidant Capacity

To assess the release of TA from the coatings and their antioxidant capacity (Figure 5), coatings with thick 30 min TA layers were prepared, while the number of bilayers was increased from 3 to 5. In addition, two extra conditions with TA as the last layer (TA-PAA)_4_-TA, were included to determine how the final layer in the LbL process affects coating stability and antioxidant capacity. The TA monolayer was included as a control, and the coating time of the TA monolayer was set to 4 h to match the whole coating time of the LbL assembly.

**Figure 5:**
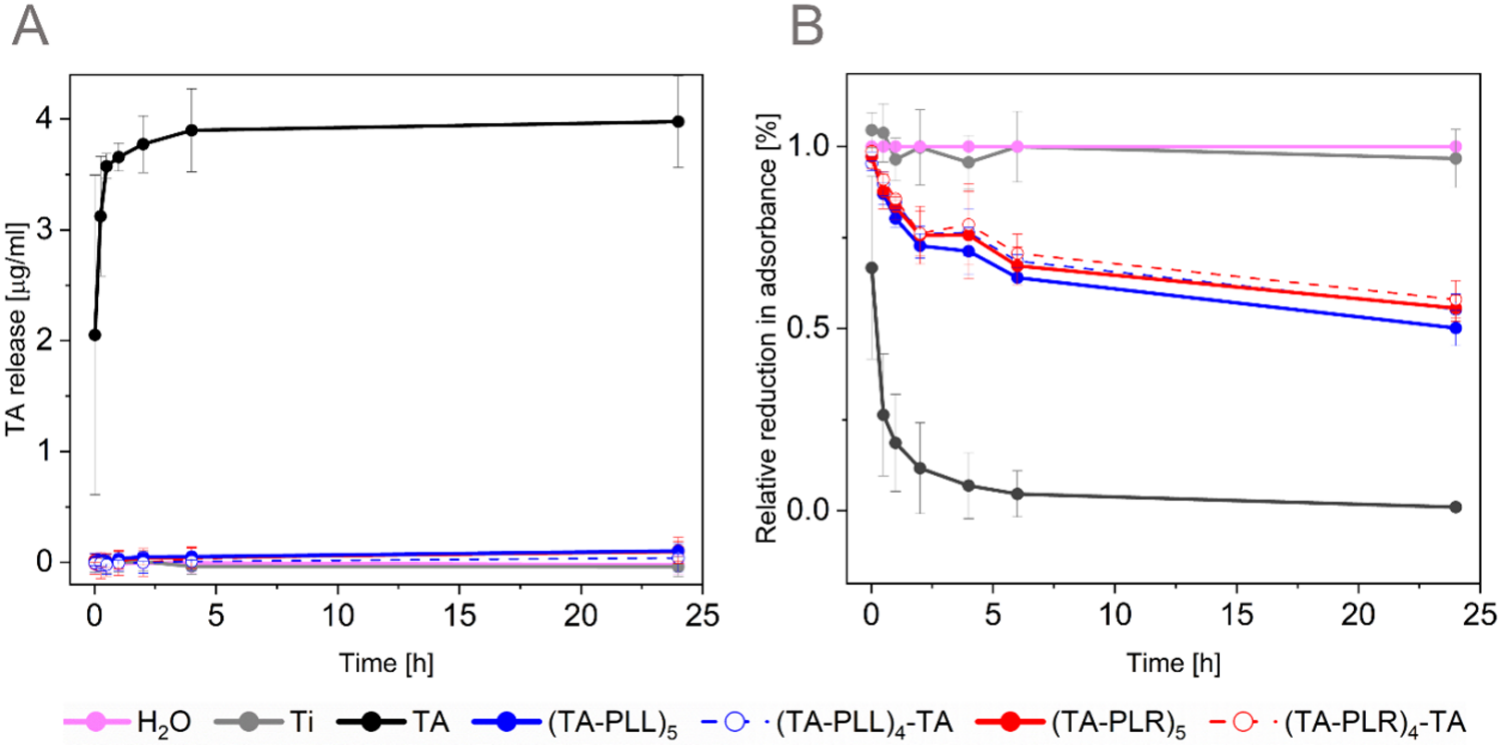
Release of TA (A) and antioxidant capacity of LbL coatings (B). TA layer deposition time was 30 min. Coatings with a TA top layer are abbreviated as (TA-PLL)_4_-TA or (TA-PLR)_4_-TA, while coatings with a PAA top layer are abbreviated as (TA-PLL)_5_ or (TA-PLR)_5_. Results are presented as mean ± SD (*n* = 3).

To assess the stability of the coatings (Figure 5), the concentration of TA or its polyphenolic degradation products released from the coatings was measured using a Prussian Blue assay. In the case of the TA monolayer, an immediate release was detected in the first 45 min. The release continued at a slower rate, reaching the maximum after 6 h. In contrast, no polyphenol release was observed from the samples containing PAAs, independently of the final layer. It can be hypothesized that the presence of PAA prevented TA from being released because of the physical interactions between TA and PAA. Release data for 7 days of immersion in H_2_O (Figure S7) showed that no TA was released from the coatings, irrespective of the topmost coating layer. The TA monolayer reached a stable signal after 6 h, which was maintained over time.

Considering the TA antioxidant properties and that it was not released, the antioxidant capacity of TA in the coatings was further investigated. For this, the ABTS assay was used (Figure 5B). The TA monolayer exhibited high antioxidant capacity during the initial minutes, reducing almost 80% of ABTS radicals in the first 45 min, with almost 100% radicals scavenged by 6 h. This coincided with the maximum TA release. The addition of PAAs reduced the antioxidant capacity of TA. However, despite TA not being released, the LbL coatings retained antioxidant activity, reaching up to 50% of the antioxidant capacity of the TA monolayer. Sustained antioxidant capacity and hindrance of TA release may decrease unwanted pro-oxidant effects, which can cause DNA damage and decrease cell viability. Strong interaction between PAAs and OH groups of TA may explain the reduction of antioxidant capacity of the multilayers.^71^

### 3.4 Cell Viability and Morphology

To evaluate biological activity of the multilayer coatings, we cultured human gingival fibroblasts on top of the coatings for 24 h. Along with multilayers, we also tested disks coated with monolayers of TA, PLL, and PLR. As shown in Figure 6A, cell viability remained high (>75%), except for fibroblasts grown on titanium coated with PLR monolayer showing compromised viability (32 ± 27%). These findings aligned with the cytotoxicity analysis of the examined coatings (Figure 6B), determined by checking the integrity of hGFs membranes and the related release of lactate dehydrogenase (LDH) into the culture medium. All tested surfaces exhibited low cytotoxicity, except the PLR monolayer, with 44 ± 22% increase in cytotoxicity. This may be attributed to PLR’s cell-penetrating properties that are higher than for PLL.^72^ Specifically, the positively charged arginine residues can interact with the negatively charged components on the cell membrane, leading to membrane destabilization, pore formation, and, in severe cases, cellular lysis.^72^

**Figure 6:**
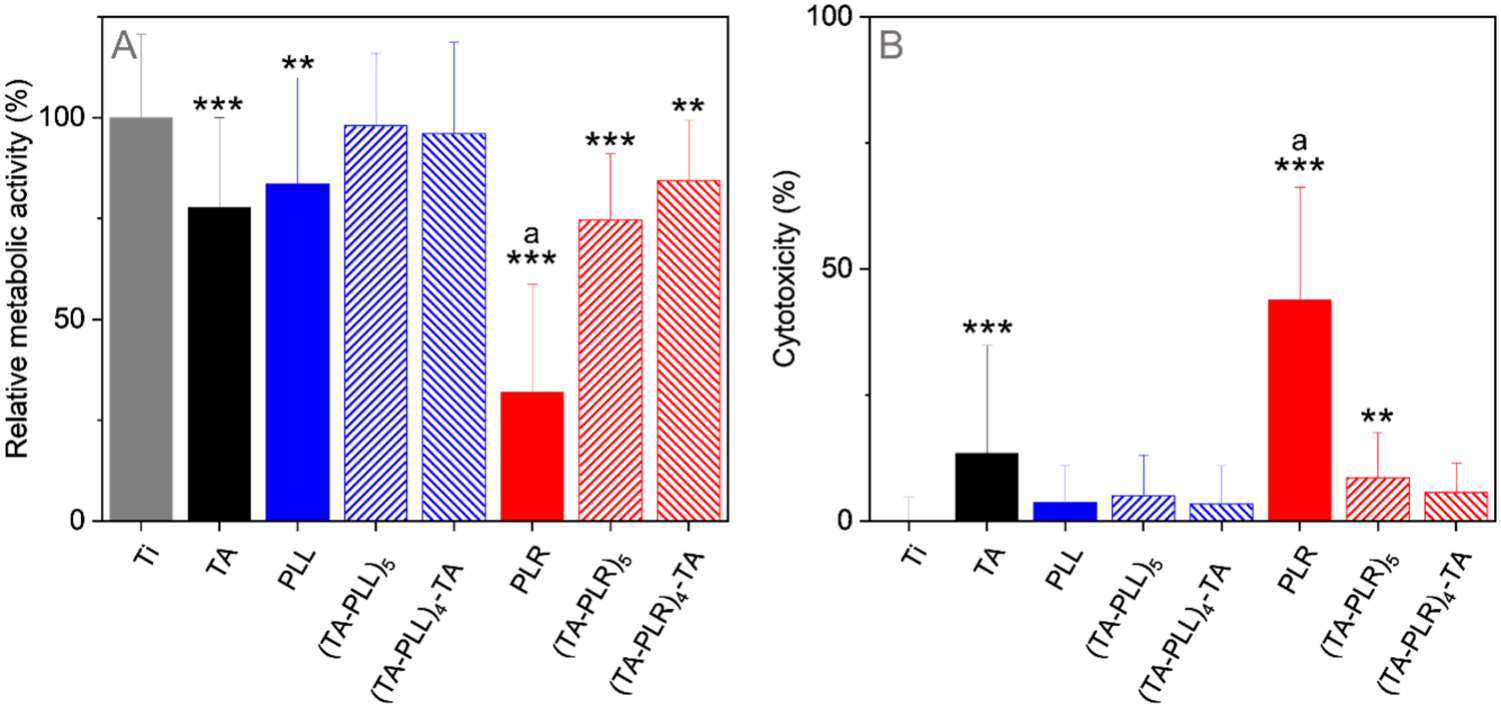
Comparative analysis of metabolic activity (A) and acute cytotoxicity (B) for hGFs cultured for 24 h on unmodified (control) and modified titanium disks. Cytotoxicity was measured by the percentage of lactate dehydrogenase (LDH) release from the cells. Asterisks indicate statistically significant differences compared to the Ti group, *p* value <0.05 (*), *p* value <0.01 (**), *p* value <0.001 (***). Letter above the bar indicates statistically significant difference from all other groups, *p* value <0.001. Mean ± SD (*n* = 3).

An enhancement in cell viability was observed when culturing the cells on TA-PLL-modified titanium, compared to the TA monolayer. Lower cell viability on the TA monolayer can be attributed to a higher rate of TA release, as shown in Figure 5A. Increased TA concentration in the culture medium may affect the surrounding cell function and viability due to its pro-oxidant properties.^73^ However, a comparable amount of TA incorporated into LbL coatings, resulting in a substantial reduction of TA release, consequently improved the biocompatibility of the coating.

As can be seen from Figure 6, cell viability was lower on TA-PLR than on TA-PLL coatings. According to our results, TA-PLR coatings had higher thicknesses of both TA and PLR layers and were almost twice the thickness of TA-PLL coatings. Furthermore, TA-PLR coatings contained more PLR compared to the amount of PLL in the TA-PLL coatings. Considering the high cytotoxicity of PLR alone, lower viability on TA-PLR coatings can be partially attributed to higher concentrations of both TA and PLR.

Variation of a terminating layer (TA or PAA) did not significantly influence the cell viability. When PLL and PLR were employed as terminating layers in LbL coatings, they were probably involved in hydrogen bonding with the underlying TA layer. This interaction strengthened the bonds between layers, resulting in a more rigid top layer, having implications for the mechanical properties of the film. It has been previously shown that increased rigidity of LbL coatings may at least partially explain the enhanced cell growth and spreading.^74^ Moreover, the hydrogen bond formation between PLL or PLR and the hydroxyl groups of TA alters the accessibility of positively charged amino groups in these PAAs (Figure S8). It is possible, therefore, that their engagement in these intermolecular interactions makes them less available for interactions with biological molecules or cellular components, further diminishing cytotoxic effects.

Figure 7 shows that cells demonstrated surface-dependent behavior, as reflected by differences in morphology, distribution, and focal adhesion (FA) formation. On uncoated titanium, fibroblasts were evenly distributed and exhibited a classic spindle-like shape with elongated bodies and well-defined nuclei. The cells were aligned uniformly, displayed a well-organized F-actin cytoskeleton with stress fibers oriented along the cell’s long axis, and formed multiple FAs, indicating strong adhesion to the underlying substrate. On the TA monolayer, cells were less confluent and formed tight clusters. In addition, they were smaller and adopted irregular shapes with numerous filopodia enriched with actin filaments and FAs. On the PLL monolayer, fibroblasts maintained a morphology similar to that on unmodified titanium, displaying a comparable number of FAs. However, these cells were less spread and not as uniformly aligned, indicating subtle changes in cell behavior due to the PLL coating. On the contrary, cells on the PLR monolayer entirely inhibited cell adhesion and spreading. Cells exhibited ameboid or rounded shapes, with a less pronounced F-actin cytoskeleton and no development of FAs. Furthermore, most cells appeared translucent, indicating potential cellular distress or compromised viability. These observations support our results showing increased cytotoxicity along with reduced metabolic activity of the cells cultured on PLR. Notably, fibroblasts exhibited enhanced morphology and adhesion on LbL-coated substrates compared to monolayers. Despite being smaller and less elongated, these cells developed numerous FAs, indicating a robust and stable attachment to the surfaces. Cell appearance was similar on all tested LbL coatings, independently of the terminating layer or the type of PAA used. Therefore, our results demonstrate that combining PAA and TA into LbL multilayer coatings significantly enhances cytocompatibility compared with using these molecules individually.

**Figure 7:**
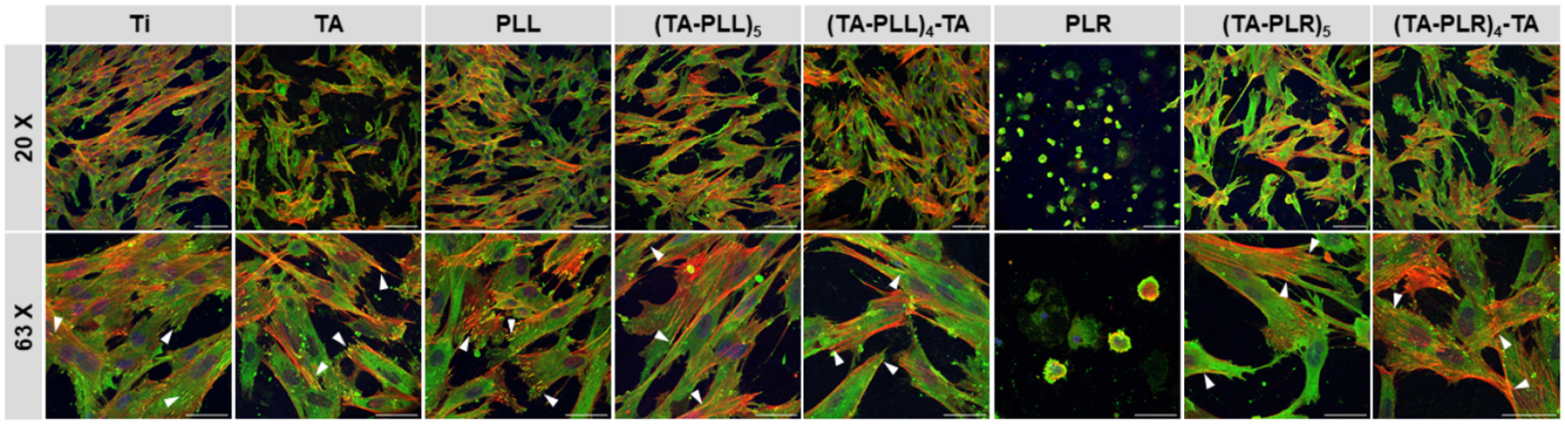
Fluorescence microscopy images of hGFs cultivated on unmodified and modified titanium disks for 24 h. Cells were stained for F-actin (red), nuclear DNA (blue), and vinculin (green). Arrows indicate focal adhesions. Scale bar is 100 and 50 μm for 20 and 63× magnification, respectively.

## 4 Conclusions

All of the studied TA-PAA multilayers were found to be stable in aqueous solutions at physiological pH. TA layer thickness could be easily controlled due to silicate-TA cross-linking, allowing coatings with variable amounts of TA to be formed between the deposited PAA layers. The dissipation and thickness of the deposited TA-PLR bilayers were higher compared to TA-PLL due to the structural and chemical differences between the two PAAs. The interaction between TA and PAA was further investigated by using FTIR and UV–vis, and was shown to be noncovalent, likely driven by electrostatic forces and hydrogen bonding. In the presence of PAA, TA was not released from the coatings, in contrast to TA monolayers. TA-PAA coatings maintained the antioxidant capacity of TA irrespective of the topmost layer, despite TA not being released from the LbL coatings. In general, all tested LbL coatings showed high cell viability, which was higher for TA-PLL than TA-PLR. The characteristic elongated shape of fibroblasts and the formation of multiple FAs was observed on both TA-PLL and TA-PLR multilayers, indicating high cell adhesion to the coatings. Therefore, the presented approach of combining TA and PAAs to LbL coatings can be used as a multifunctional surface modification strategy to improve host tissue response to biomedical implants. However, the mechanical properties and long-term stability in biological environments should be further studied.

## Supplementary Material



## Notes

Interaction between the multilayers and differently charged nanoparticles used from zeta potential measurement; TA 60 min coating deposition and 2 bilayers; QCM-D plots at working pH 7.8, comparison between TA deposition versus TA-PAA layer-by-layer deposition; coating thickness measured using AFM; representative AFM profiles; release of TA over 7 days; surface zeta potential of the deposited coatings (PDF)
