# Supplementary material for: Multifunctional Tannic Acid and Polyamino Acid Layer-by-Layer Coatings for Tailored Implant Surfaces
Source: ACS Biomater Sci Eng. 2025 Aug 19;11(9):5376–88. doi: 10.1021/acsbiomaterials.5c00832 (PMC12421510; doi:10.1021/acsbiomaterials.5c00832)
Supplement: Supplementary file 1 [file ab5c00832_si_001.pdf]

Supporting information

Multifunctional tannic acid and polyamino acid layer-by-layer coatings for tailored implant surfaces

Enrique Oreja[1], Daria Zaytseva-Zotova[1], Agnes Rogala[1], Alejandro Barrantes[2], Phillip B. Messersmith[3,4], Hanna Tiainen[1,*]

[1]Department of Biomaterials, University of Oslo, PO Box 1109 Blindern 0317 Oslo, Norway

[2]Oral Research Laboratory, University of Oslo, PO Box 1109 Blindern 0317 Oslo, Norway

[3]Departments of Bioengineering and Materials Science and Engineering, University of California, Berkeley, 210 Hearst Mining Building, CA 94720 Berkeley, USA

[4]Materials Sciences Division, Lawrence Berkeley National Laboratory, 1 Cyclotron Road Berkeley, CA 94720 Berkeley, USA

Corresponding author, E-mail: hanna.tiainen@odont.uio.no

Abbreviations

Tannic acid (TA), polyamino acid (PAA), poly-L-lysine (PLL), poly-L-arginine (PLR) and the subindex 2,3 and 5 correspond to the number of bilayers. Layer-by-layer (LbL)

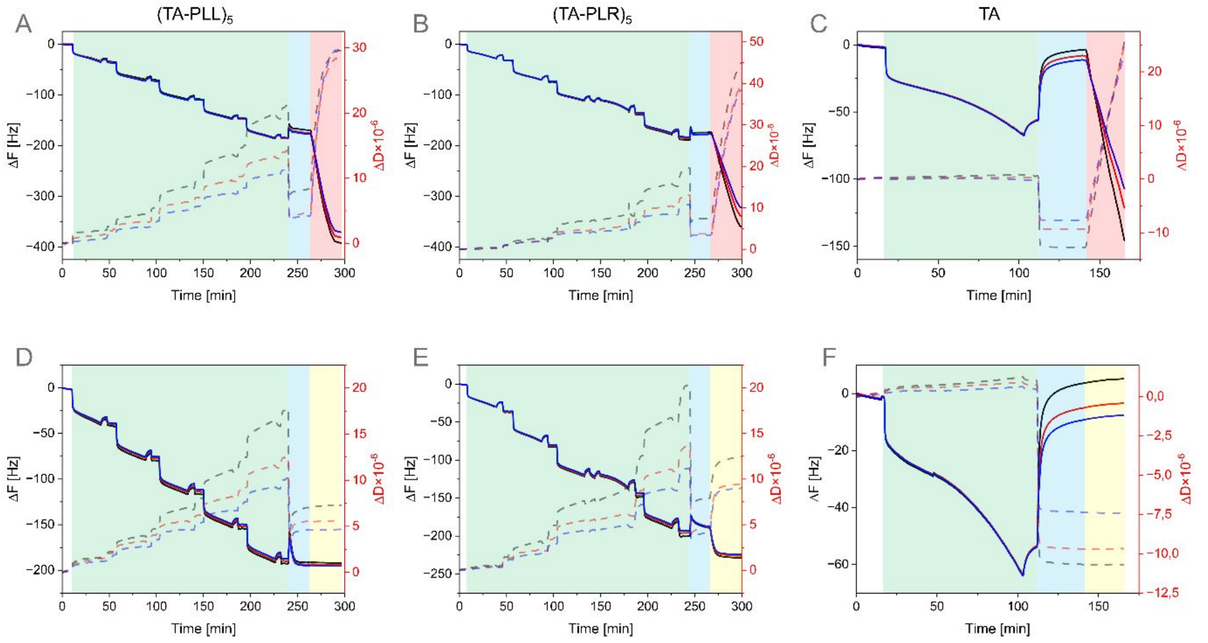

Figure S1. Representative frequency and dissipation shifts for 3rd, 5th and 7th harmonics (n) showing the interaction between the formed multilayers and the charged nanoparticles used for zeta potential measurements. Coating deposition onto Ti sensor (green) of $(TA\text{-}PLL)_5$ (A, D), $(TA\text{-}PLR)_5$ (B, E) and TA monolayer (C, F). Blue colored part of the graph indicates change in buffer from coating buffer pH 6,8 to buffer containing 2 mM sodium phosphates and 10 mM NaCl. Two differently charged tracer nanoparticles were used, positively charged NR_3^+ (red) and negatively charged COO^- (yellow).

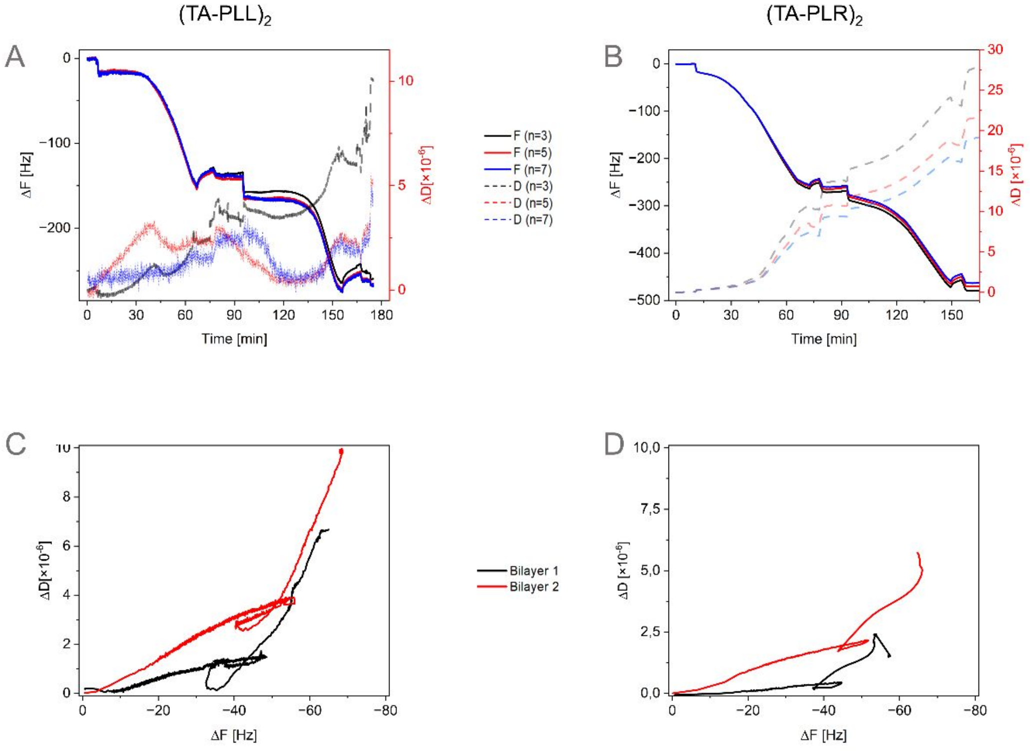

Figure S2. Representative frequency and dissipation shifts for 3rd, 5th and 7th harmonics (n) as a function of time for TA-PAA multilayer deposition at pH 6,8. Continuous monitoring of frequency and dissipation changes for $(TA\text{-}PLL)_2$ (A) and $(TA\text{-}PLR)_2$ (B). TA layers were deposited for 60 minutes. Data was replotted as $\Delta D/\Delta F$ to obtain qualitative information about the viscoelastic properties of the coating (C, D).

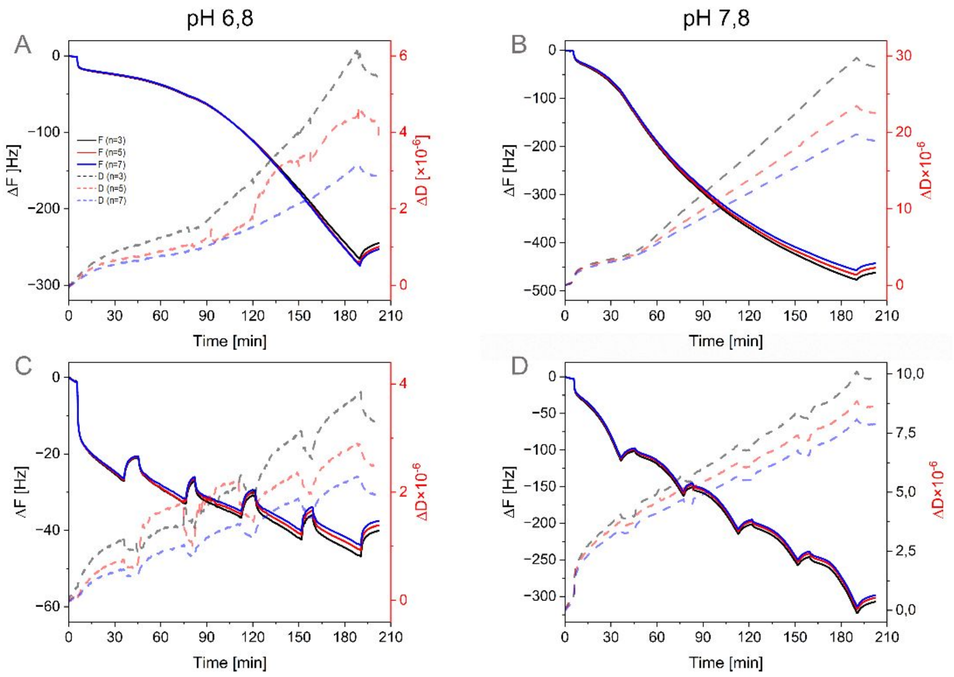

Figure S3. Representative frequency and dissipation shifts for 3rd, 5th and 7th harmonics (n) for TA deposition at pH 6,8 and pH 7,8. Comparison between continuous deposition of TA (A, B) vs LbL (C, D). The effect was studied in non-oxidative conditions (A, C) and oxidative conditions (B, D) by varying the pH from 6,8 to 7,8. LbL consisted of depositing of 5 TA layers for 30 minutes, including a washing step with buffer for 10 minutes in between each layer. The continuous deposition time matched the LbL coating time, including a washing step at the end.

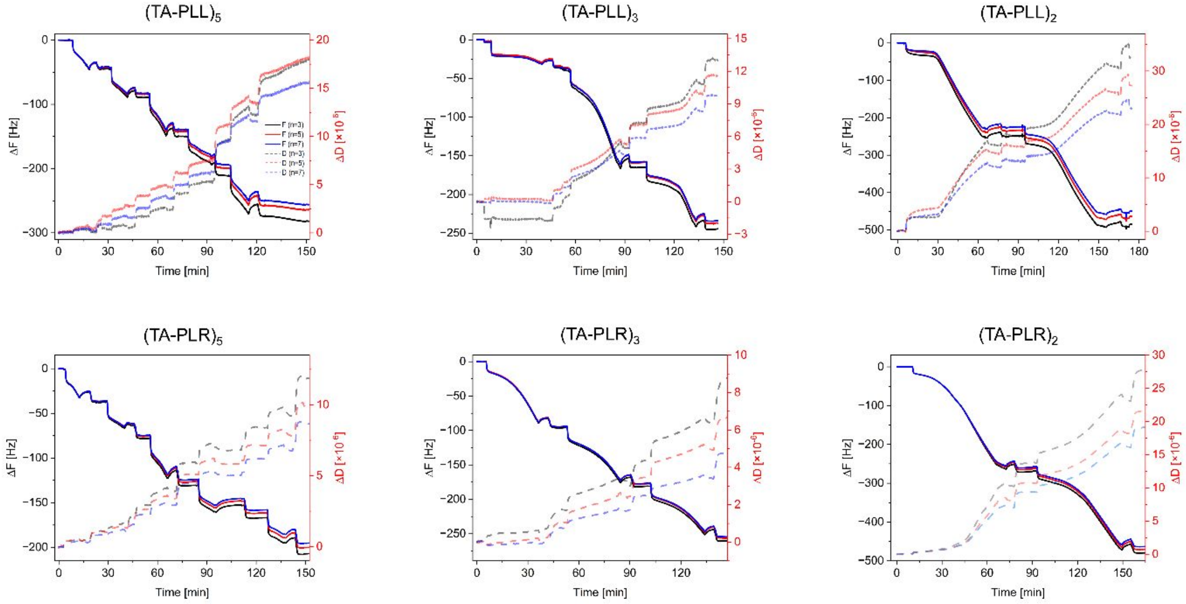

Figure S4. Representative frequency and dissipation shifts for 3rd, 5th and 7th harmonics (n) as a function of time for TA-PAA deposition at pH 7,8. Comparison of multilayer coatings with TA deposited for 10, 30 and 60 minutes. For each condition, the coating formation followed similar trend as observed at pH 6,8.

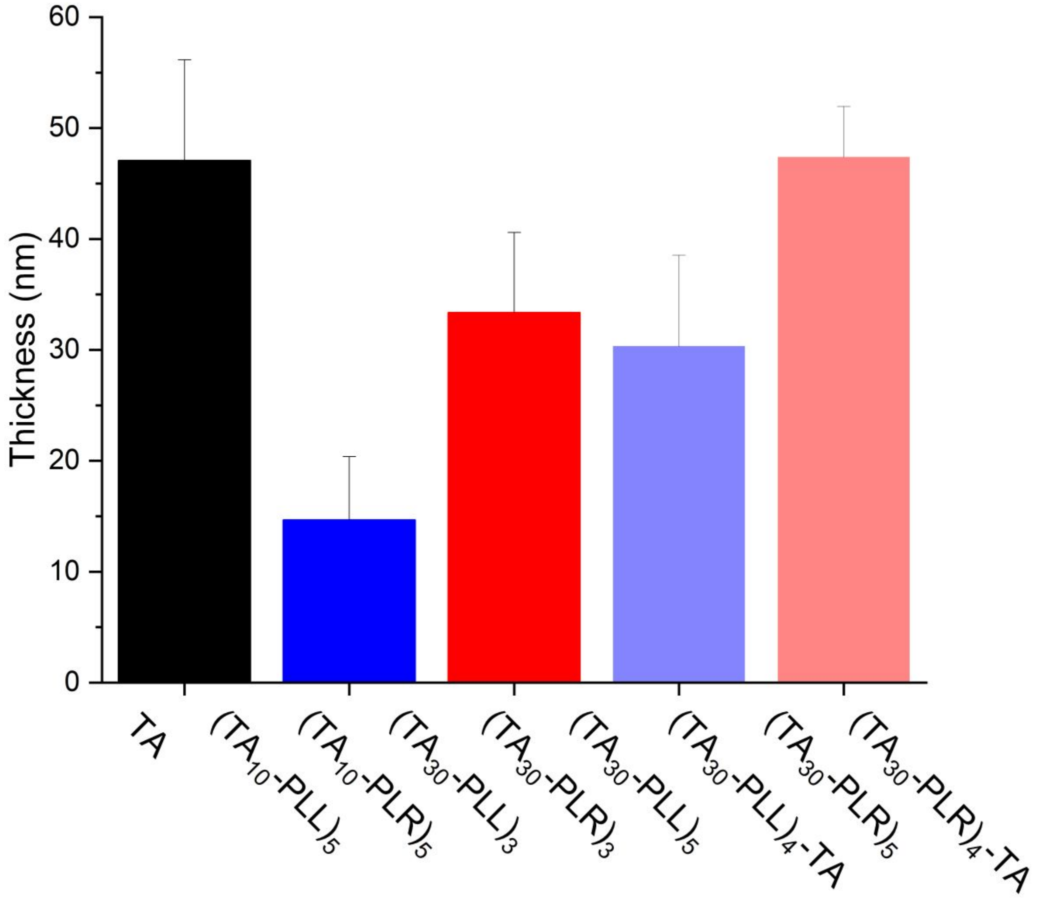

Figure S5. Thickness measured by AFM. Coatings were deposited Si wafers as described in the manuscript. Half of the silicon wafer was covered with silicon during the coating process to use it as a reference from flat surface. The thickness was determined by measuring the heigh difference in the obtained profiles presented in **Figure S6.** Each value consists of n=3 profiles with 10-pixel width. Results are presented as mean ± standard deviation.

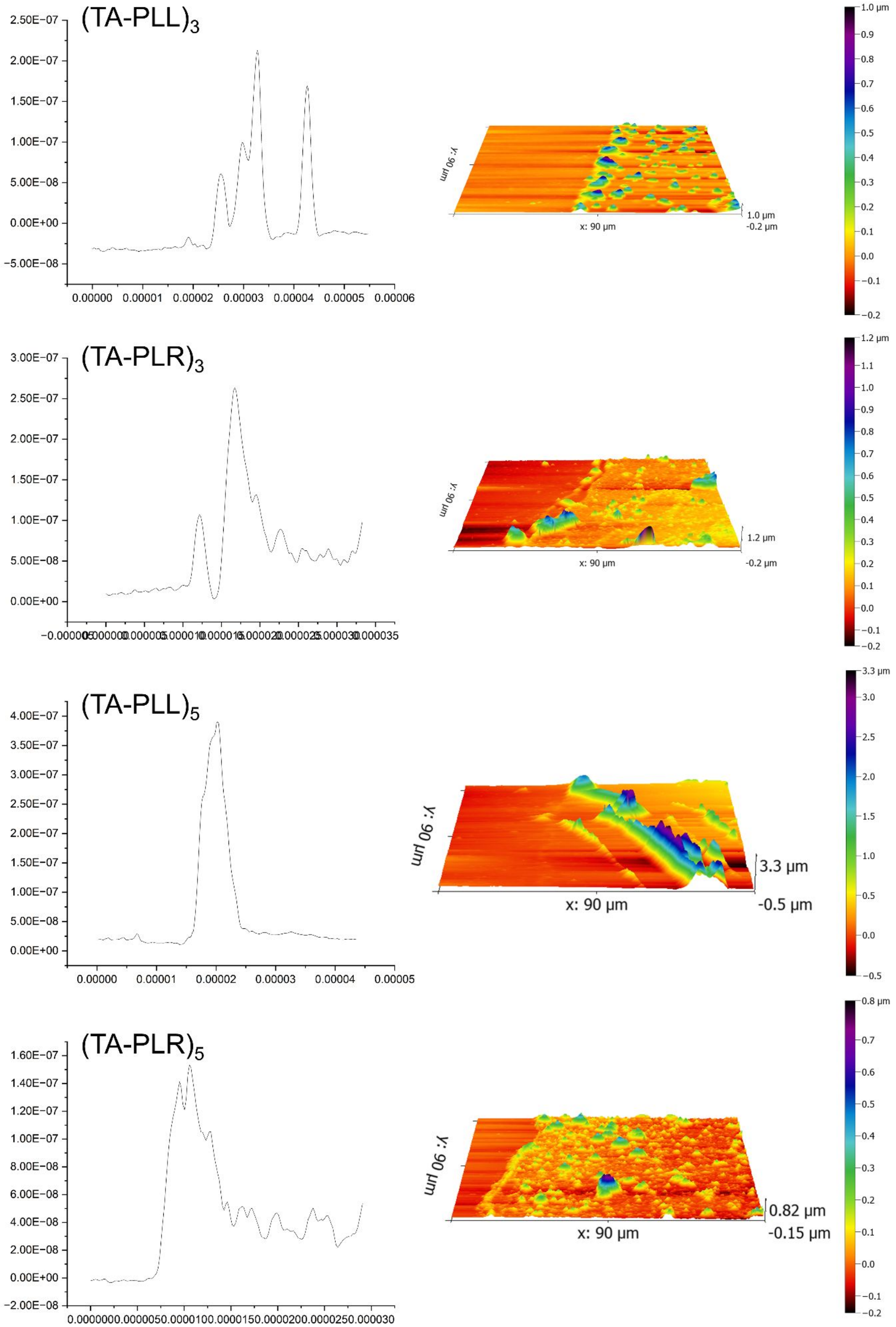

Figure S6. Representative AFM profile images. Coatings were deposited Si wafers as described in the manuscript. Half of the silicon wafer was covered with silicon during the coating process to use it as a reference from flat surface.

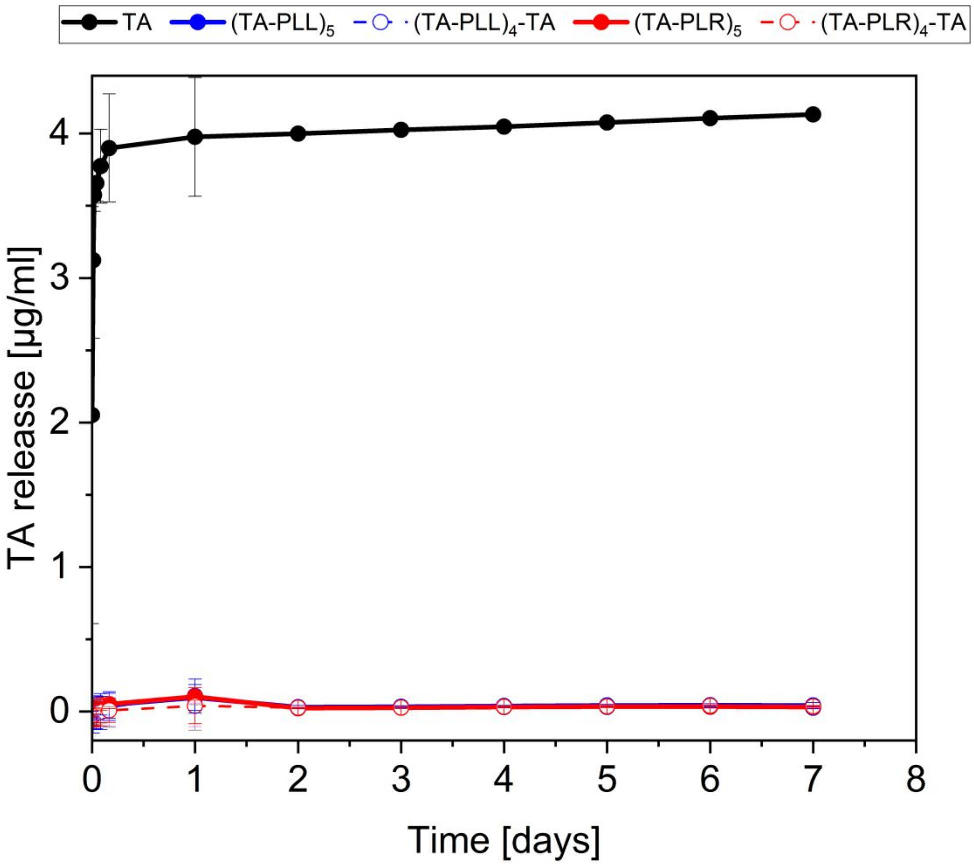

Figure S7. **TA release from the coatings after immersion in H_2O for 7 days.** The coatings were deposited on titanium coins as described in the manuscript. After 7 days immersion in H_2O Prussian blue assay was performed. No TA release was observed for any of the TA-PAA LbL coatings during the 7-day immersion period irrespective of the topmost coating layer, whereas TA release reached a stable signal already after 6 hours. The slight increase in the released amount of TA from day 1 to day 7 can be attributed to evaporation of the solvent over time, despite the samples being sealed with parafilm.

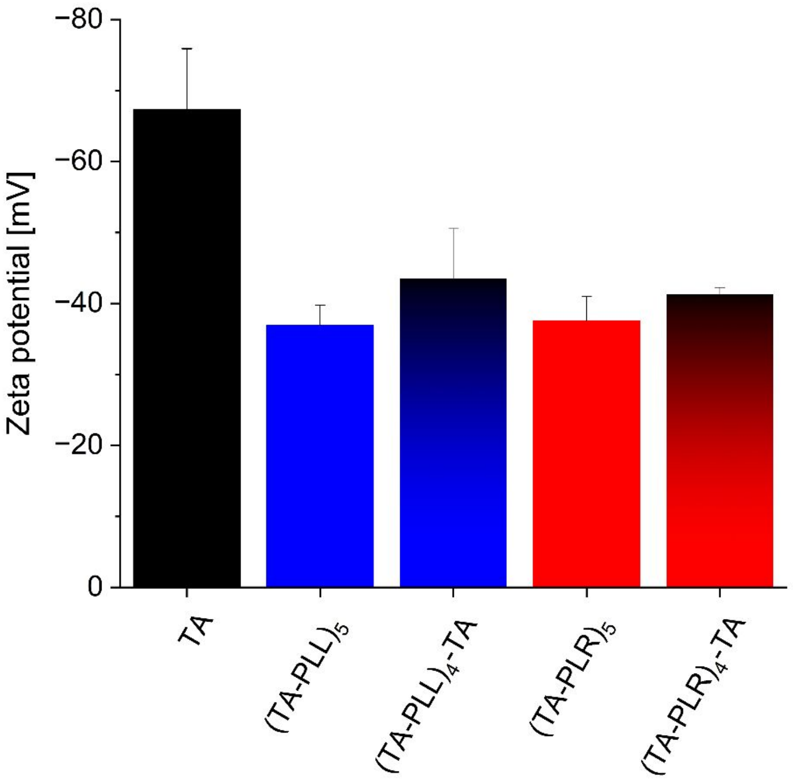

Figure S8. **Surface zeta potential of coated silicon wafers.** The coatings were measured in buffer containing 2 mM sodium phosphates and 10 mM NaCl and negatively charged nanoparticles. Measurements were conducted at a conductivity of 0.5 mS/cm. Results are presented as an average of three individual experiments (n=3).
